# Report of the Committee on the Means of Preserving Milk, and on the Influence of Pregnancy and Menstruation on the Composition and Nutritive Qualities of That Fluid

**Published:** 1856-01

**Authors:** N. S. Davis

**Affiliations:** From the Transactions of the American Medical Association


### THE NORTH-WESTERN

### MEDICAL AND SURGICAL JOURNAL.

(NEW SERIES.)

VOL. V. JANUARY, 1856. NO. 1.

### ORIGINAL COMMUNICATIONS.

ARTICLE I.—*Report of the Committee on the Means of Preserving Milk, and on the Influence of Pregnancy and Menstruation on the Composition and Nutritive Qualities of that Fluid.* Prepared for the American Medical Association, Annual Session of May, 1855. By N. S. Davis, M.D., &c. From the Transactions of the American Medical Association.

The general subject assigned to your Committee requires consideration under three distinct heads, viz: First, the means capable of preserving milk free from injurious changes in its composition for an indefinite length of time; Second, the changes which the milk of the female undergoes during the period of menstruation; and, Third, the changes which occur in the same fluid in those cases where pregnancy supervenes while lactation is still going on.

If we keep in mind the whole period of human life, from the tender period of infancy to old age, we shall readily perceive that Milk is not only one of the most important, but also one of the most essential articles of diet in the possession of man. It is the only article provided by nature for the first period of life, and for which the genius of man has never succeeded in devising a substitute. Embracing in its composition all the elements necessary for the nutrition of the various structures of the body, and in a form so bland and easily absorbed as to be adapted to the most delicate condition of the digestive apparatus, it is often no less valuable to the invalid and the convalescent than to the infant. In this one fluid we have, in the form of organic matter, *casein*, *oils*, and *sugar;* and as inorganic matter, phosphates of lime, magnesia, and iron, chlorides of sodium, potassium, and soda; and sulphur. All these ingredients are either dissolved or suspended in water, in nearly the following proportion, viz:

| | |
|---|---|
| Water, - - - | 87.7 |
| Organic matter, - | 10.8 |
| Inorganic matter, - | 1.5 |
| | 100.0 |

The only ingredient not in a state of complete solution is the oil or butter, which exists in the form of minute globules, lighter than the water, and consequently disposed to rise to the surface whenever the milk is allowed to remain at rest. The casein, which is one of the most abundant ingredients, closely resembles albumen in its chemical composition, and like it, liable to undergo decomposition or change by exposure to heat or air in a moist state. But of all the constituents of milk, the sugar is the quickest to undergo changes by time and temperature. Chemically identical with grape sugar, it is very easily converted into lactic acid by a process of fermentation. At an ordinary summer temperature, from six to twelve hours is sufficient to effect the change, or at least to begin it. In the natural condition of fresh milk, the soda is in combination with the casein, and is the means of rendering the latter soluble in the water. No sooner, however, is lactic acid generated from the decomposition of the sugar, than it unites with the soda, leaving the casein free, and consequently insoluble, and in the form of white coagulated masses. These changes constitute the process, familiarly known as the "*souring*" of milk. By this very brief glance at the composition of milk, we see in the number and nature of its ingredients, and the feeble affinity by which some of them are held together, the reason why it so speedily undergoes important changes when exposed to the influence of time and temperature. In rural districts, where animals from which fresh milk may be obtained are abundant, the necessity of preserving the milk for the purpose of rendering it less liable to change, is not felt. But on ships at sea, during long journeys over uninhabited regions of country, and in large cities, the difficulty of obtaining pure and fresh milk is very great; so much so, indeed, as to be not merely a source of discomfort, but of greatly increased sickness and mortality, especially during the first two years of life.

The very high ratio of mortality among children in all our large cities is a fact familiar to all. And though many causes may combine to produce this result, yet long continued and careful observation has satisfied your committee, that among them there are none more prolific of evil than the defective supply of pure milk. The dairies which are kept within the environs of large cities, and on which the citizens chiefly depend, are subject generally to so much confinement and unwholesome food, that both the cows and their milk become diseased and impure, even when no adulterations are resorted to by the dealers.

When railroads were first constructed, extending from the cities into rich agricultural districts, it was anticipated that the rapidity of transportation would ensure a constant and abundant supply of the article from the rich pastures of the country. But a little experience was sufficient to demonstrate the falsity of these anticipations. For however short the time required for transportation, the additional time used in transferring the milk from the farm house to the cars, from the latter to the general depot, and from thence to individual customers scattered over a large city, accompanied at each step by more or less agitation, is such, that through all the warm season, even with the greatest possible care, the milk is so diluted by the ice used to keep it cool, and so near the full change called *souring*, that it is totally unfit for the nourishment of young children and adults with sensitive organs of digestion. We speak on this subject from much personal experience and observation. Hence there could scarcely be a more important improvement, or one which would contribute more to the health and happiness of men than the discovery of some economical and easy method of rendering milk incapable of undergoing change by time and temperature, while at the same time its nutritive qualities and easy digestibility remained unaltered.

During the last half century various attempts have been made to accomplish this result, but until quite recently with very little success. If they have in some intances succeeded in preserving the milk a long time fit for use, it has been either by depriving it of some of its valuable ingredients, or by adding other substances to such an extent as to materially alter its qualities. Thus the plan of M. Appert, consisted in boiling the milk in an open vessel until it was reduced one half in bulk, frequently skimming it, and finally adding the yolk of one egg to each quart of the concentrated milk. It is then to be kept in bottles. An article of "preserved milk," sometimes found in the market in the form of paste, is thought by a committee of the New York Academy of Medicine to have been prepared by a process somewhat similar to the method of M. Appert; it containing a considerable quantity of albumen. It is easy to perceive that the boiling and skimming process of M. Appert, and the subsequent addition of albumen, or the yolk of eggs, materially alters the quality of the milk and renders it objectionable.

The most successful method yet devised for the preservation of the nutritive qualities and ingredients of milk, in a form at once permanent and portable, is that of Mr. Samuel T. Blatchford of Dutchess County, New York. By his method the water of the milk is evaporated and all its other constituents obtained in a dry and solid state, mixed with a certain proportion of white sugar. When prepared for the market it is in the form of square or oblong tablets, covered with tinfoil, each weighing one pound. In this form it is very firm and dry, and has been carried on ships through almost every parallel of latitude and longitude, and kept more than twelve months without change; it being when re-dissolved in water possessed of all the constituents and qualities of fresh milk, with the simple addition of sugar. Its capability of being kept without unfavorable change through any reasonable period of time, and under exposure to a temperature equal to the warmest seasons and climates, provided it be kept dry, has been fully tested. Your committee have been unable to detect any change in the qualities of specimens, carelessly left in a box in the office, during all the past summer.

The same preparation of milk is also found in the market put up in cans, instead of solid tablets. In the cans it is in the form of a dry, granular powder, and is more readily soluble in water than the tablets. The latter for use requires to be grated fine, or pulverized, and may then be readily dissolved either in cold or tepid water; a pound of the solid tablet being sufficient to convert five pints of water into a good quality of fresh milk. During the summer of 1854, a committee of the New York Academy of Medicine, consisting of Drs. J. H. Griscom, John R. Van Kleek, Benj. Drake, W. N. Blakeman, John Shanks, Joseph M. Smith, Saml. A. Purdy, A. H. White, James Stewart and James M. Minor, visited the establishment of Mr. Blatchford in Dutchess County.

In their subsequent report to the Academy the committee use the following language, viz:

"It (the process) has been fully subjected to our critical examination; we have traced the milk of the rich pasturage of Dutchess County, from the udder to its final conversion into the solid tablet; and we find it, in all its stages and appliances, to be based upon a thorough knowledge of the chemistry and dynamic tendencies of the natural fluid. It is not within our province, nor would it be proper here, to detail the steps of this operation; and it will suffice for this to state that the article called '*Solidified Milk*,' obtained from that locality, and presented to us for examination, is nothing but the solid constituents of pure milk, combined with a little less than an equal part, by weight, of white sugar; *that it contains no other foreign substance;* that the various solids of the original fluid are preserved intact, even the butter globules being unbroken; that it is readily and perfectly soluble in water, and when it is so disolved in proper proportion, it is in fact milk as it was secreted from the cow, with the sole exception of the sugar which accompanies it, that the only medicinal or culinary operations in which ordinary milk is required, and this article cannot be used, are those in which sugar is inadmissible, while on the other hand, whenever sugar is required in connection with milk, they are here found together."—In another part of the same report, it is said that, "the only objection, besides that of the presence of sugar, which can be made to it is an empyreumatic flavor somewhat similar to that of boiled milk, which it receives in the process of manufacture. This varies in degree, but is much less distinct when the solution is made with cold than with hot water, and in the preparation of custards, puddings, arrow-root, wine-whey, ice-creams, &c., in all of which your committee have practically tested it, it disappears."—In regard to the change of constituents the committee further observe: "There is no loss of any nutritive material, a fact of which we can always be assured, for the article cannot be produced except from *fresh* milk, as any change in the character of the original fluid, either by spontaneous decomposition, or otherwise, must spoil the result. So any dilution of the fluid, by water, must only lengthen the process of manufacture and thereby endanger the issue."

These opinions, expressed by so able a committee, after a personal examination of the process, and a careful investigation of the whole subject, are sufficient, doubtless, to satisfy the members of this Association that the "*solidified milk*" is all that the manufacturer claims it to be; and that the great desideratum in regard to the preservation and portableness of that important article of nourishment has been fully attained by Mr. Blatchford.

But having, previously to the reception of the report from which I have been quoting, subjected the "*solidified milk*" to a rigid investigation, I will briefly add the results of my own observations and experiments.

When perfectly dissolved in water, in the proportion of three ounces to the pint, and examined under the microscope, no difference could be detected between it and fresh milk, except the presence of sugar in the former. Annexed are accurate sketches of both, copied from the field under the microscope magnifying 800 diameters. Repeated chemical analysis showed clearly the presence of all the solid constituents of pure milk in their natural proportion, with the addition of sugar and a small excess of soda. It is true, the New York committee assert, "that it contains *no other foreign* substance" than sugar, but unless my examinations have been altogether deceptive, there is a larger amount of *soda* than belongs naturally in the milk. Its quantity, however, is not sufficient to constitute the least objection, or to be noticed in any of the purposes to which the milk may be applied. On the contrary, alkali being a natural constituent both of the milk and the blood, can hardly act as a foreign ingredient in the limited quantity which I suppose to exist in the substance under consideration. Having obtained none but the most satisfactory results from the microscope and chemical examinations, I next directed my attention to its use as an article of food, both for the sick and the well. I caused three children under two years of age, to be fed almost exclusively on the solidified milk dissolved in water, for two weeks in succession. The results were in every respect as favorable as when the same children were fed on the best quality of cows' milk. I also embraced some opportunities to use the article for the nourishment of young infants, who were deprived of milk from the breast of the mother, and who were found incapable of being nourished on such milk as was furnished by the milk-men of the city. The following is one of the cases of this kind:

Mrs. S. was confined in her first labor, on the 16th day of September, 1854. She was delivered of a healthy male child, without any untoward symptoms; but four days after was attacked with symptoms of puerpural peritonitis, from which she recovered in about ten days, but without any flow of milk sufficient to nourish the child.

During the sickness of the mother, and for ten days subsequent thereto, efforts were made to nourish the child on the milk furnished by the milk-man of the city; but it was often rejected by the stomach in a *sour state*, and the child emaciated rapidly, its bowels became disordered, the mouth covered with *thrush*, and two or three abscesses made their appearance in the cellular tissue of the neck and shoulders. Seeing the child in this unpleasant condition, I furnished the family a tablet of the solidified milk, with the request that it should be dissolved in water as required for use, in the proportion of three ounces to the pint of water, and given to the child *ad libitum;* at the same time withholding all other nourishment, and prescribing no remedies except a powder of alum and white sugar to heal the mouth. So soon as this change was made in the diet of the child, it began rapidly to improve. It ceased to vomit, the bowels became more regular, and in a few days it began perceptibly to increase in flesh.

It continued to be fed almost exclusively on the solidified milk for several weeks, during which it remained well, regained a good degree of flesh, and grew finely. The parents soon after left the city, and the child passed beyond the reach of my observation. To detail other cases would extend my report to an unnecessary length. It is sufficient to observe, that all my experiments, calculated to test the nutritive qualities and digestibility of the article, have led to highly satisfactory results.

From the observations thus far made, I have been able to discover only three objections to the substance under consideration. The first is that it contains an extra quantity of sugar. As an article of nourishment, and for most culinary purposes, this objection would have no weight; bnt to use with tea and coffee, it would be disagreeable to many. The second objection is the empyreumatic flavor spoken of by the committee of the New York Academy of Medicine, and which is certainly quite perceptible when used alone, or with tea and coffee. But this flavor is disliked by a comparatively small number of persons only, repeated use would doubtless soon remove the *dislike* even with them. The third objection has reference to the price; which, if I am correctly informed, is twenty-five cents per pound at retail, being equivalent to about *four* cents a *pint* for pure milk. On the other hand, the solidified milk of Mr. Blatchford's manufacture, possesses some advantages of importance. Its capability of perfect preservation enables us to procure it by the box as we would sugar, thereby saving all the trouble of being waited upon by a milk-man once or twice a-day; and what is of still greater importance, it may be dissolved and ready for use in just such quantities as needed, in a few minutes, thereby enabling those who use it to have at all times a *fresh* article. Again, it may not only be diluted like ordinary milk to any desirable degree, but it may also be dissolved in such proportion as to render the solution much more rich in nutritive constituents than ordinary milk; thereby furnishing more nourishment in a small quantity of fluid, a quality of importance in some conditions of the digestive organs. From the investigations I have been able, thus far, to make, I have been led to regard the following conclusions as essentially correct, viz:

1st, That in the "solidified milk" manufactured by Mr. Samuel T. Blatchford, of New York, there is preserved all the solid and nutritive constituents of fresh and pure milk, in such a state as to be capable of remaining unchanged for years, if kept free from moisture.

2d, That the solid constituents of the milk so preserved, retain their natural combinations and qualities so perfectly, that when dissolved in water, in proper proportion, the solution possesses all the chemical, microscopical, physical, and nutritive qualities of fresh and pure milk from the cow, modified only by the presence of an excess of white sugar and a trifling proportion of soda.

3d, That the article may be used, not only safely, but advantageously, for all the purposes to which milk is applied, except those in which sugar is positively inapplicable.

Its portableness, its solubility, and consequent capacity to furnish at all times a solution perfectly fresh, and of any required degree of richness, renders it especially applicable to the nourishment of young children and invalids. And hence, your committee do not hesitate to recommend it as a reliable substitute for milk procured by the methods in ordinary use, not only on ships at sea, and during long journeys overland, but also as an article for general use in all our large cities.

A tablet of the "solidified milk," manufactured by Mr. Blatchford, about ten months since, is herewith presented to the Association for the inspection of its members.

In regard to the remaining topics referred to your committee for investigation, viz: the influences of menstruation and pregnancy on the composition and qualities of milk, I am not able to make a satisfactory report. Although I have gathered some facts of interest, yet it is easy to perceive that the opportunities for a careful and thorough investigation of these topics would not be numerous, in the practice of any one physician during the short period of one year. Should the Association deem these topics of sufficient importance to continue the committee, I shall be happy to pursue the investigation until more satisfactory results are obtained.

All of which is respectfully submitted.

